# Electrostatic Interaction as a Key Modulator of Na^+^,K^+^-ATPase Function

**DOI:** 10.1007/s00232-026-00382-7

**Published:** 2026-05-26

**Authors:** Shadreen Fairuz, Zhitong Li, Amy Gorman, Flemming Cornelius, Ronald J. Clarke

**Affiliations:** 1 School of Chemistry, University of Sydney, Sydney, NSW 2006 Australia; 2 Department of Biomedicine, University of Aarhus, Aarhus C, DK-8000 Denmark; 3 The University of Sydney Nano Institute, Sydney, NSW 2006 Australia

**Keywords:** Calcium, Magnesium, P-type ATPase, Solid-supported-membrane, Ionic strength, Electrophysiology

## Abstract

The Na^+^,K^+^-ATPase is an integral membrane protein present in all animal plasma membranes. It uses the energy of ATP hydrolysis to pump 3 Na^+^ ions per ATP hydrolysed from the cell cytoplasm into the extracellular fluid in exchange for 2 K^+^ ions. The Na^+^ electrochemical potential gradient it produces is used as an energy source to drive all animal secondary transporters, for example, in nutrient reabsorption in the kidney. In this synthesis of new and previously published results, it is shown that the rate of the mammalian enzyme’s rate-determining E2 → E1 conformational change, during which K^+^ ions are released to the cytoplasm, is strongly dependent on an electrostatic interaction in the E2 state. This electrostatic interaction must be broken to allow the enzyme to convert to the E1 state and allow Na^+^ pumping. The strength of the interaction depends on ionic strength and, even more strongly, on the concentration of divalent metal ions, i.e., Ca^2+^ or Mg^2+^. A likely candidate for the interaction is between the protein’s positively-charged lysine-rich N-terminus and the negatively-charged membrane cytoplasmic surface. Comparison of the Ca^2+^ and Mg^2+^ dissociation constants measured with the physiological levels of the two ions suggests that Mg^2+^, but not Ca^2+^, could play a regulatory role for the Na^+^,K^+^-ATPase.

**Graphical Abstract:**

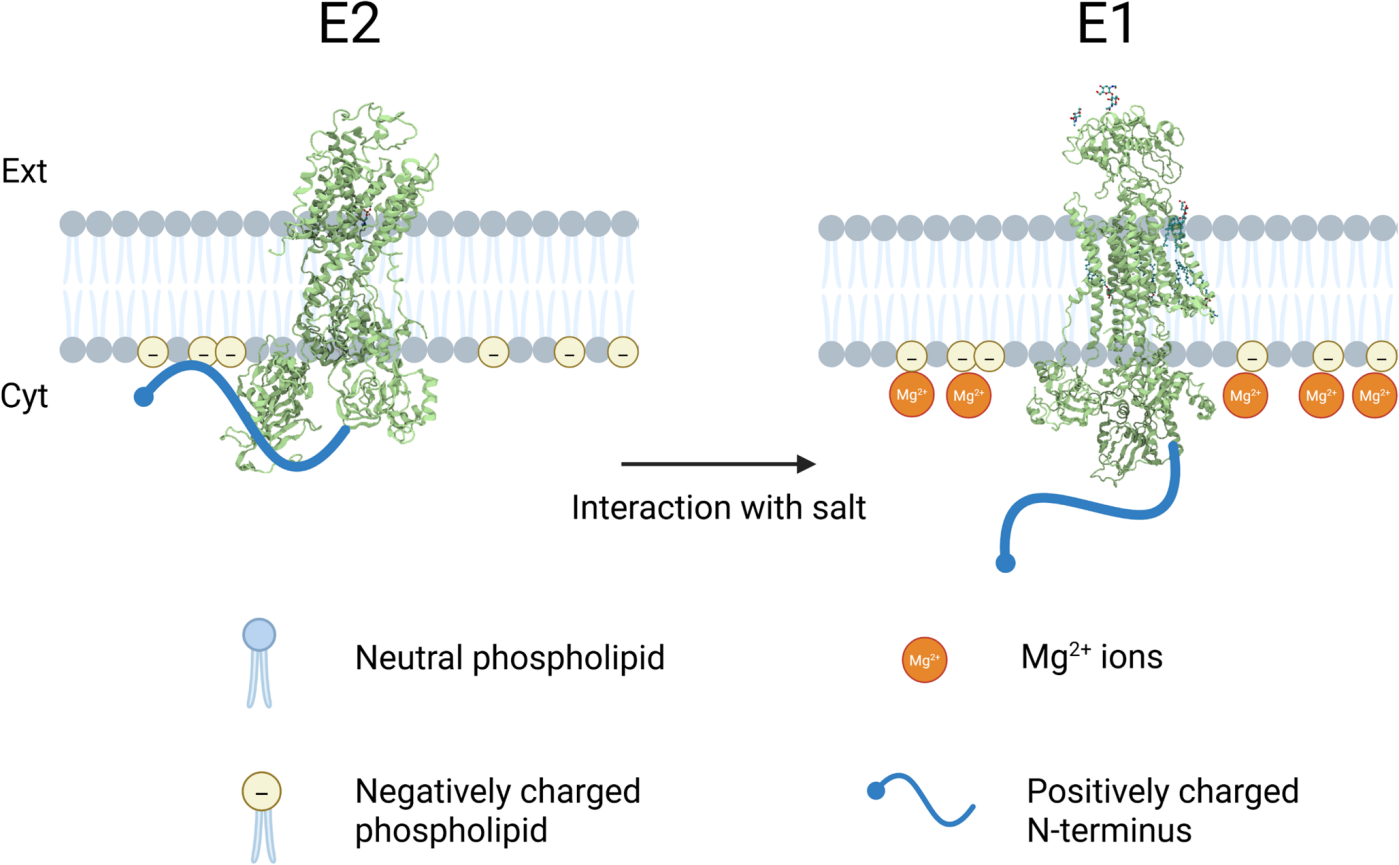

## Introduction

The Na^+^,K^+^-ATPase is a member of the P-type ATPase family. Other prominent members of this family include the sarcoplasmic reticulum and plasma membrane Ca^2+^-ATPases and the H^+^,K^+^-ATPase of the stomach mucosa (Møller et al. 1996). The Na^+^,K^+^-ATPase is a transmembrane protein expressed in all animal cells. It utilizes energy from ATP hydrolysis to transport three Na^+^ ions out of and two K^+^ ions into the cell per ATP molecule hydrolyzed. The electrochemical potential gradients of these ions which the Na^+^,K^+^-ATPase thus maintains across the cell membrane are essential to fundamental cell functions such as solute transport and cell volume regulation (Kaplan 2002). The protein consists of a catalytic α-subunit with a large cytoplasmic domain, a smaller β-subunit with a small extracellular domain and, in various tissues, an even smaller subunit with a single membrane-spanning α helix, in the case of kidney cells the so-called γ-subunit.

**Fig. 1:**
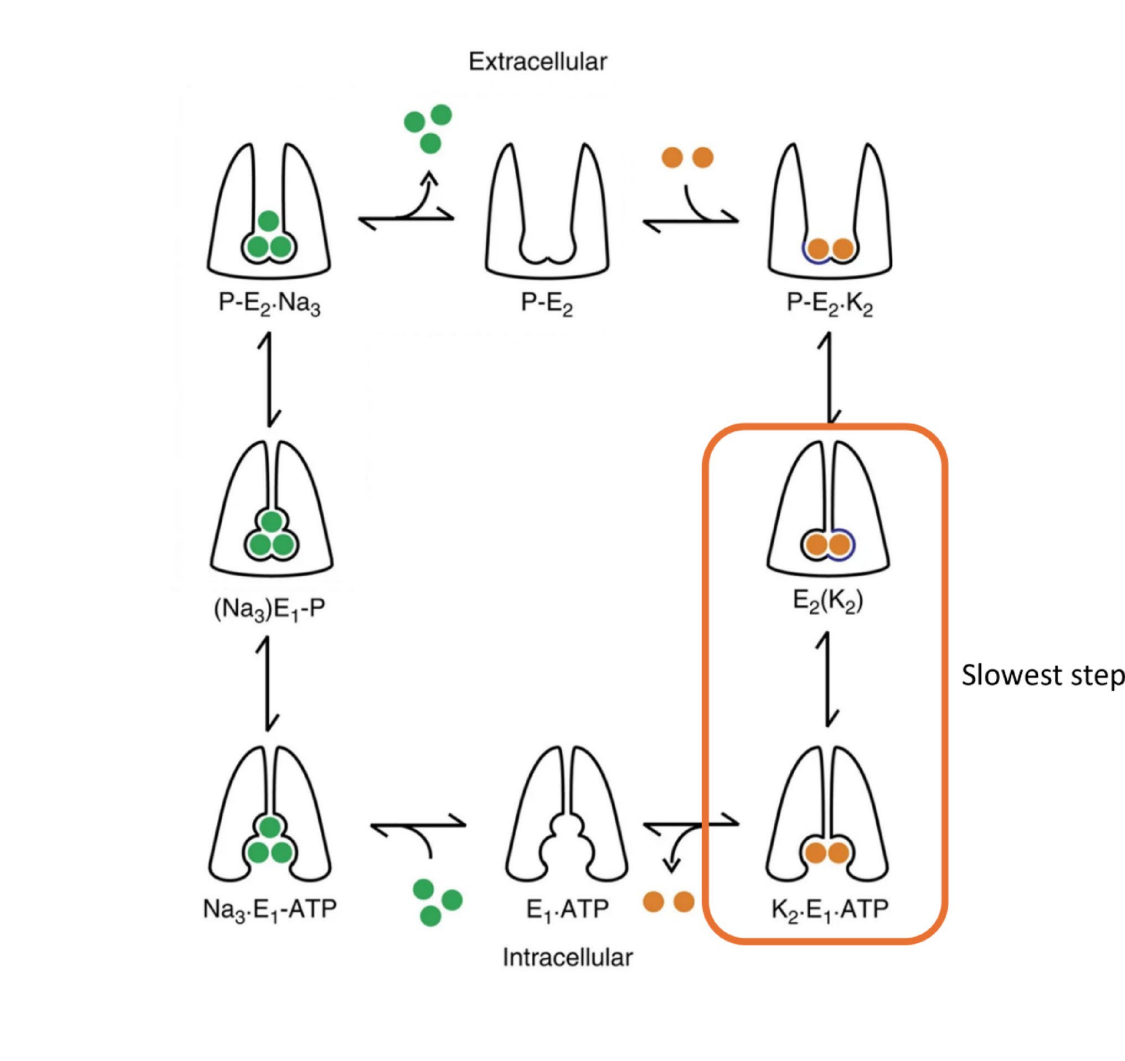
The Albers-Post or E1-E2 reaction cycle, describing the sequence of partial reactions that the Na^+^,K^+^-ATPase undergoes. Na^+^ ions are represented by green balls and K^+^ ions by orange. The rectangular box highlights the reaction on which this article concentrates, the E2 → E1 conformational transition of unphosphorylated Na^+^,K^+^-ATPase. Modified from Gadsby et al. (2012) with permission.

Kinetic studies on purified Na^+^,K^+^-ATPase from different mammalian tissue sources have established that a conformational change of the unphosphorylated enzyme associated with the release of K^+^ ions to the cytoplasmic medium is the slowest step of the mammalian enzyme’s ion pumping cycle, i.e., the transition E2(K^+^)_2_ → E1 + 2 K^+^ (Humphrey et al. 2002) (see Fig. 1). Modulation of the kinetics of this reaction step would, therefore, be expected to have a significant effect on the overall ion pumping turnover and it would, thus, be a prime location for regulation of the enzyme’s activity. Crystal structural studies provide an explanation for the slow nature of this reaction step, because it is associated with significant movement of the cytoplasmic domains of the catalytic α-subunit, in particular opening of the protein’s cytoplasmic headpieces as the N (nucleotide binding) domain becomes detached and moves away from the A (actuator) domain (Kanai et al. 2023).

Measurements using buffer cations to vary the ionic strength, together with the probe eosin, which binds to the protein’s ATP binding site (Hossain et al. 2020) and is sensitive to the Na^+^,K^+^-ATPase’s E2-E1 conformational change (Skou and Esmann 1981, 1983), have shown that the conformational state is very dependent on the ionic strength (Jiang et al. 2017). The observed changes in the probe’s fluorescence excitation spectrum reached half-saturation at an ionic strength of approximately 5 mM. This indicates that electrostatic interactions, such as a salt bridge, are present in the E2 state and that screening and breaking of these interactions by salt induces the transition into the E1 state. This is also consistent with the kinetic effects of salts on the E2-E1 transition measured by Humphrey et al. (2002) using the fluorescent probe RH421, which is thought to respond to changes in the degree of interaction of the Na^+^,K^+^-ATPase’s N-terminus with the cytoplasmic surface of the surrounding membrane accompanying the E2-E1 conformational transition (Garcia et al. 2017). The results of Humphrey et al. (2002) showed an increase in the observed rate constant of the E2-E1 transition with increasing salt concentration. This is characteristic for an interaction of salt with the reactant, i.e., E2. If salt were inducing the transition by stabilizing E1, the reverse behaviour would have been expected, i.e., a decrease in the observed rate constant with increasing salt concentration.

**Fig. 2:**
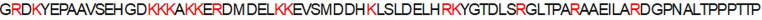
Amino acid sequence in FASTA format of the N-terminus of the α_1_ subunit of the Na^+^,K^+^-ATPase from *Sus scrofa* (pig) up to the beginning of the first transmembrane helix (Xiong et al. 2023). Positively-charged basic residues are highlighted in red, i.e., lysine (K) and arginine (R)

**Fig. 3:**
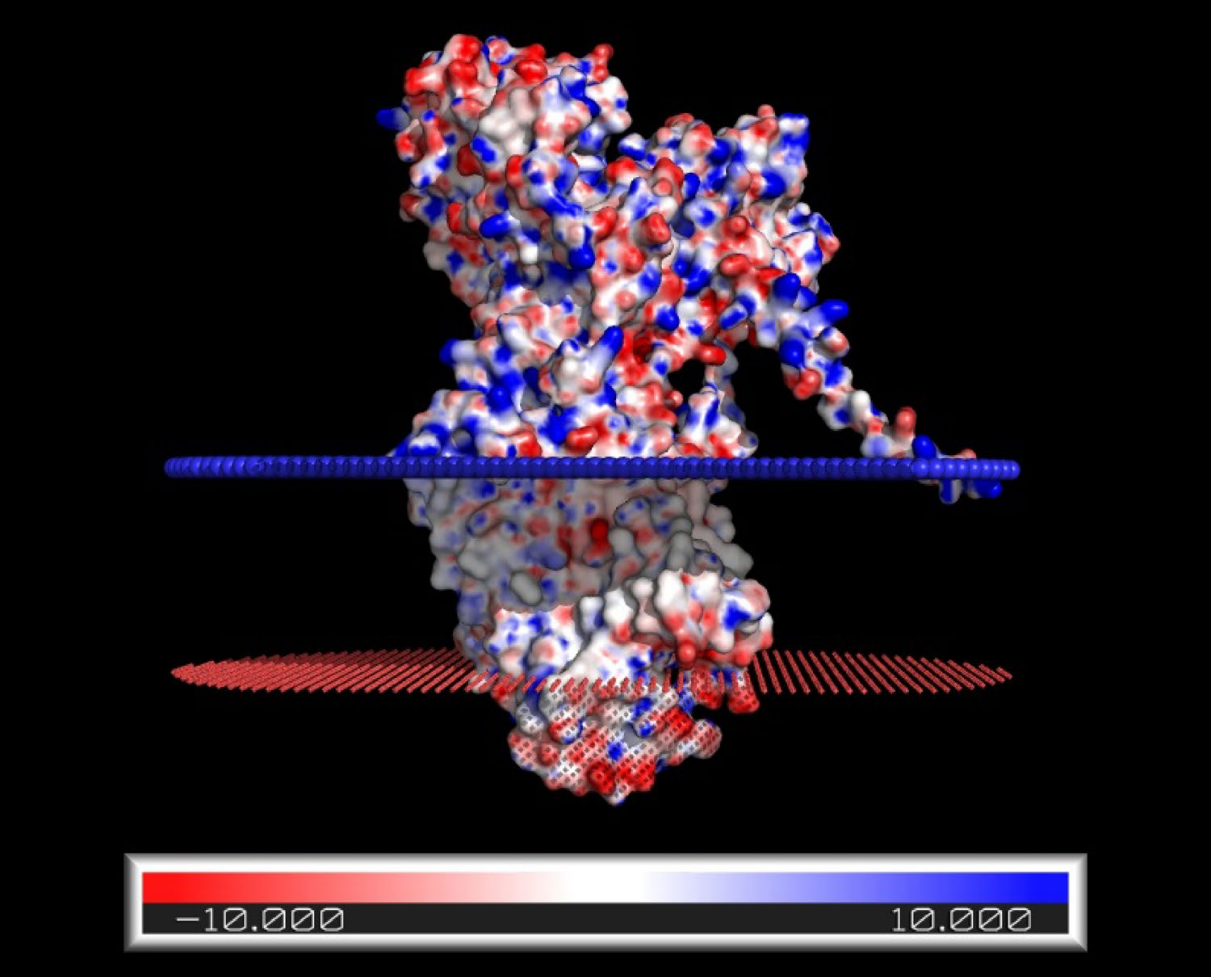
Tertiary structure analysis of pig, *Sus scrofa*, Na^+^,K^+^-ATPase α_1_ subunit

According to crystal structures published so far, there are no observable salt bridges that stabilize the E2 conformation (Kanai et al. 2023), only hydrogen bonds. But, in contrast to a salt bridge, hydrogen bonding is not universally weakened by ionic strength regardless of the ion used to increase it (Urbic 2014). Therefore, the ionic strength results of Jiang et al. (2017), which were obtained using buffer cations of varying structures, can only be explained by a salt-bridge type interaction in a part of the protein which so far hasn’t been resolved in the crystal structures. Based on the knowledge that the lysine-rich N-terminus (see Figs. 2 and 3) of the α-subunit moves significantly during the E2-E1 transition (Jørgensen and Collins 1986; Jørgensen and Andersen 1988), that it so far could not be resolved in crystal structures because of its flexibility, that the cytoplasmic surface of the membrane is negatively charged due to the presence of phosphatidylserine, and that the electrostatic interaction must be exposed to the neighbouring aqueous solution to be sensitive to the ionic strength of the medium, it has been suggested that the electrostatic attraction stabilizing E2 is due to an interaction between the cytoplasmically exposed N-terminus and the membrane surface (Jiang et al. 2017; Lev et al. 2023). The idea that the α-subunit’s N-terminus plays a decisive part in the enzyme’s function is further supported by kinetic studies on Na^+^,K^+^-ATPase from shark (*Squalus acanthias*) rectal gland (Myers et al. 2011), which uncovered very different partial reaction kinetic behaviour to that of the mammalian enzyme. Inspection of the amino acid sequences of the shark and pig enzyme α_1_ subunits shows that the shark enzyme has a longer N-terminus, with the insertion of a block of seven residues, GKKDKID. These results, therefore, imply that the N-terminus plays an important role, not only in regulation of the Na^+^,K^+^-ATPase, but also in determining the kinetics of its ion pumping.

The AlphaFold-predicted protein was oriented relative to the membrane using the PPM 3.0 server (Lomize et al. 2022), where the bilayer is represented as a planar hydrophobic slab, with red and blue pseudo-atoms marking extracellular and intracellular boundaries, respectively. The PPM framework orients proteins without altering their structure; thus, the placement of the N-terminus within the transmembrane region likely reflects limitations of rigid-body modelling, and a transient N-terminal conformation, rather than a definitive membrane-inserted state. The surface electrostatic potentials were mapped onto the tertiary structure using the Adaptive Poisson-Boltzmann Solver and visualised in PyMOL. The surface colouring represents electrostatic potentials ranging from − 10 kT/e (red, negatively charged) to + 10 kT/e (blue, positively charged). Here *k* is Boltzmann’s constant (1.38 ⋅ 10^− 23^ J K^− 1^), *T* is the absolute temperature in K, and *e* is the electronic charge (1.6 × 10^− 19^ C). At 298 K kT/e corresponds to 26 mV. White constitutes regions with zero electrostatic surface potential. Colour depth indicates the magnitude of the electrostatic surface potential. It is important to note that the N-terminus is highly disordered, with low confidence rating of the predicted tertiary structures of this region in AlphaFold (Jumper et al. 2021). Therefore, the tertiary structure shown should be considered as a snapshot of the N-terminus conformation rather than a static structure. The protein visualization shows that the N-terminal region extends outward from the core structure and displays localised positively charged patches (blue) proximal to the cytoplasmic side of the membrane.

Here we provide experimental data indicating that the E2-E1 distribution of the Na^+^,K^+^-ATPase is, furthermore, dependent on the presence of the divalent metal ions, Ca^2+^ and Mg^2+^. The dependence is much stronger than that found previously from charge screening via ionic strength (Jiang et al. 2017), indicating a specific binding interaction, with both Ca^2+^ and Mg^2+^ promoting the conversion of the E2 state into E1. The results are consistent with a neutralisation of the negative charges of phosphatidylserine headgroups in the membrane surrounding the Na^+^,K^+^-ATPase, allowing release of the positively charged N-terminus from the membrane and thus facilitating the conversion of the enzyme into E1.

## Results

### Dependence of the E2-E1 Conformational Distribution on Ionic Strength

**Fig. 4:**
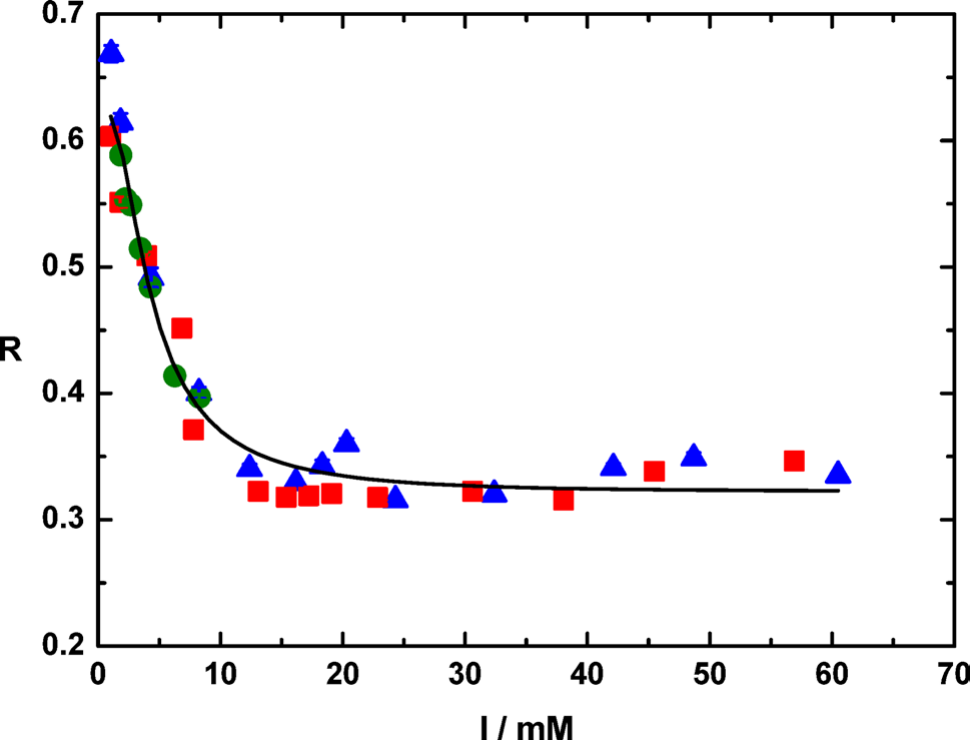
Effect of ionic strength, I, on the fluorescence ratio, R, of eosin noncovalently bound to pig kidney Na^+^,K^+^-ATPase. R is defined as the fluorescence intensity ratio using excitation wavelengths of 490 nm and 535 nm, i.e., R = F_490_/F_535_, at an emission wavelength of 550 nm. A decrease in R corresponds to a decrease in the proportion of the enzyme in the E2 conformation and hence an increase in the proportion in the E1 conformation. The points were obtained using the buffers Tris (blue triangles), imidazole (red squares) and histidine (green circles). Reproduced from Jiang et al. 2017 with permission

It has been known since the 1980s (Schuurmans Stekhoven et al. 1985) that the conformational state of the Na^+^,K^+^-ATPase is dependent on the concentration and structure of buffer compounds used. Buffer cations were said to have a “Na^+^-like” effect, shifting the enzyme into the E1 state. This seemed like a relatively obscure phenomenon, but research undertaken to understand the origin of the effect provided valuable information about the Na^+^,K^+^-ATPase mechanism. In 2001 it was shown from kinetic studies that the strength of a buffer in shifting the enzyme into the E1 state and hence accelerating phosphorylation by ATP depended on the buffer cation’s degree of protonation (Lüpfert et al. 2001). This study utilized the fluorescent probe RH421, which responds strongly to the formation of the phosphorylated E2P state. To directly measure the shift from E2 to E1 another probe has been used, eosin, which had been introduced for this purpose by Skou and Esmann (1981, 1983). Using eosin it was found that the effectiveness of buffers in promoting the E1 state increased in the order Tris > imidazole > histidine (Jiang et al. 2017). However, if the degree of protonation of the buffer was taken into account based on its pKa and the pH, and the buffer concentration was converted into an ionic strength, it was found that all buffers lay on the same curve, i.e., the effect of each buffer on the E2-E1 distribution was determined solely by the ionic strength (see Fig. 4). This result strongly suggests that conversion of the E2 state into the E1 state is controlled by the breaking of an electrostatic interaction in the E2 state, which is screened and weakened by an increase in ionic strength. However, to provide further mechanistic support for this hypothesis requires kinetic evidence to show that the ionic strength is acting on the E2 state and not on the E1 state. Thus, in principle another possibility could be that an electrostatic repulsion in the E1 state is screened by ionic strength, thus allowing the E1 state to form.

### Dependence of the Kinetics of the E2-E1 Conformational Transition on Salt

The probe RH421 can also be used to observe the kinetics of the E2 → E1 conformational transition, which causes a decrease in the fluorescence of the probe. The crucial distinction to be made here is the type of mechanism. If salt were interacting with the E1 state, i.e., with the product of the reaction, this would correspond to a lock-and-key mechanism. By stabilizing E1, the rate of the reverse reaction (E1 → E2) would be slowed and, because the reciprocal relaxation time, 1/τ, or observed rate constant, *k*_obs_, is given by the sum of the individual observed forward and backward rate constants, one would expect 1/τ to decrease with increasing salt concentration.

**Fig. 5:**
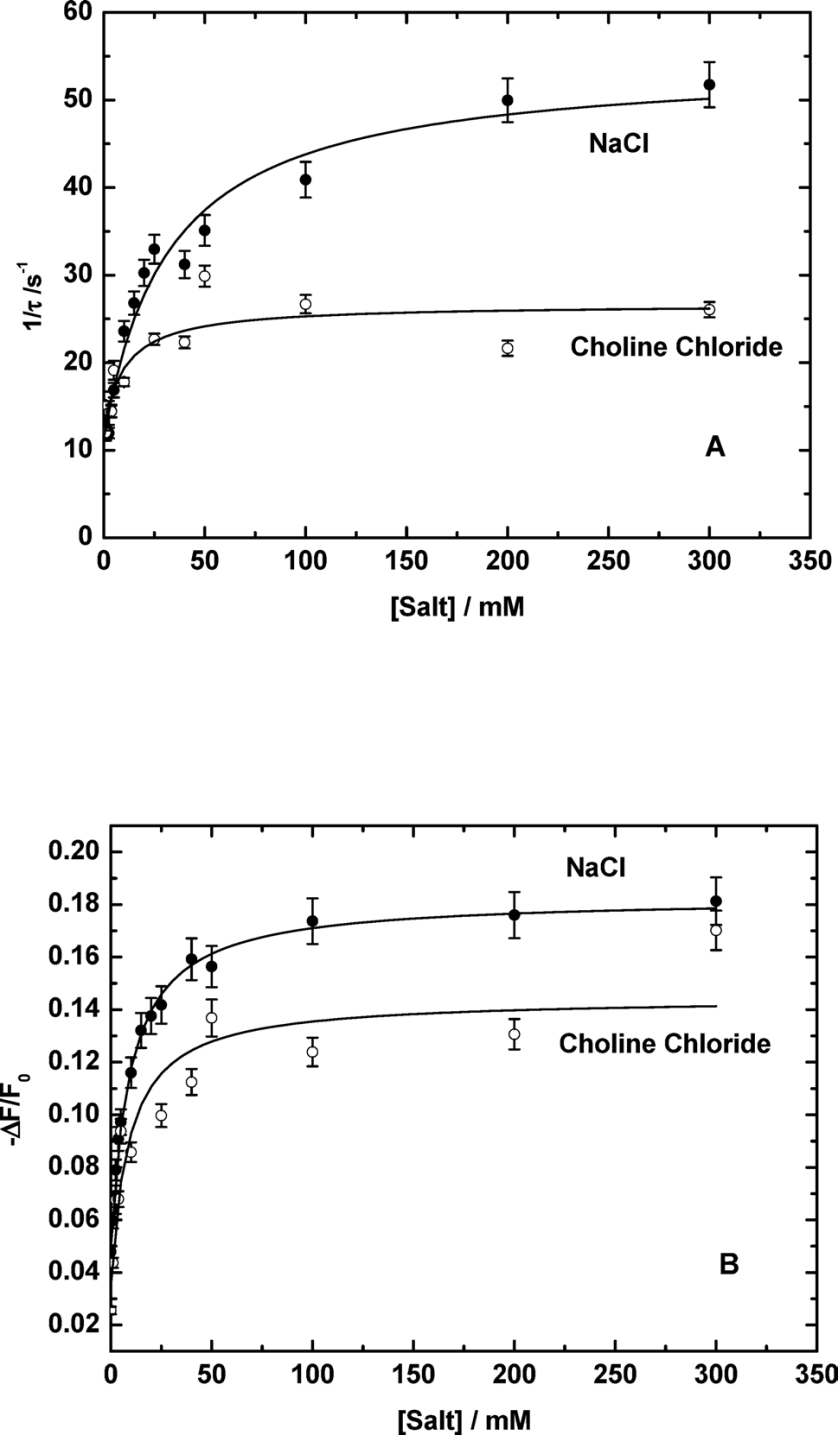
Effect of varying salt concentration, i.e. NaCl (filled circles) and choline chloride (open circles), on the reciprocal relaxation time (1/τ) (Panel A) and the relative fluorescence change (-ΔF/F_o_) (Panel B) of RH421 fluorescence transients of rabbit kidney Na^+^,K^+^-ATPase induced by mixing simultaneously with salt plus 2 mM of tris ATP. The salt concentrations given are those after mixing. Reproduced from Humphrey et al. 2002 with permission

 In contrast, if salt were interacting with E2, i.e., with the reactant in the reaction, this would correspond to an induced-fit mechanism. By destabilizing the E2 state, this would accelerate the forward reaction and one would expect an increase in 1/τ with increasing salt concentration. As one can see in Fig. 5A, the latter is what has been observed, with both NaCl and choline chloride. Therefore, the kinetic data excludes the possibility that salt favours the E1 state by weakening an electrostatic repulsion present within the E1 conformation. Therefore, the only explanation for the data shown in Figs. 4 and 5, would seem to be that the E2 → E1 transition is enabled by the weakening of an electrostatic attraction stabilizing the E2 state.

Control experiments were performed in which the fluorescence excitation spectrum of RH421 bound to dioleoylphosphatidylcholine vesicles was measured in the presence and absence of 300 mM NaCl on both sides of the membrane. In agreement with previously reported results (Clarke and Lüpfert 1999) and in contrast to experiments in the presence of a membrane-binding anion such as perchlorate (see Fig. 2, Clarke and Lüpfert 1999), it was found that NaCl caused no shift in the RH421 spectrum. The spectral changes reported here on mixing with NaCl can, therefore, confidently be attributed to a change in Na^+^,K^+^-ATPase conformation.

### Dependence of the E2-E1 Conformational Distribution on CaCl_2_ and MgCl_2_ Concentration

All of the salts that were investigated in the previous two sections were 1:1 electrolytes. It is interesting to see if the same behaviour is observed with higher order electrolytes or if there is any evidence for specific interactions, in particular with Ca^2+^ and Mg^2+^. For this purpose we have carried out titrations with CaCl_2_ and MgCl_2_ utilising the probe eosin to detect any shift in the E2/E1 distribution.

**Fig. 6:**
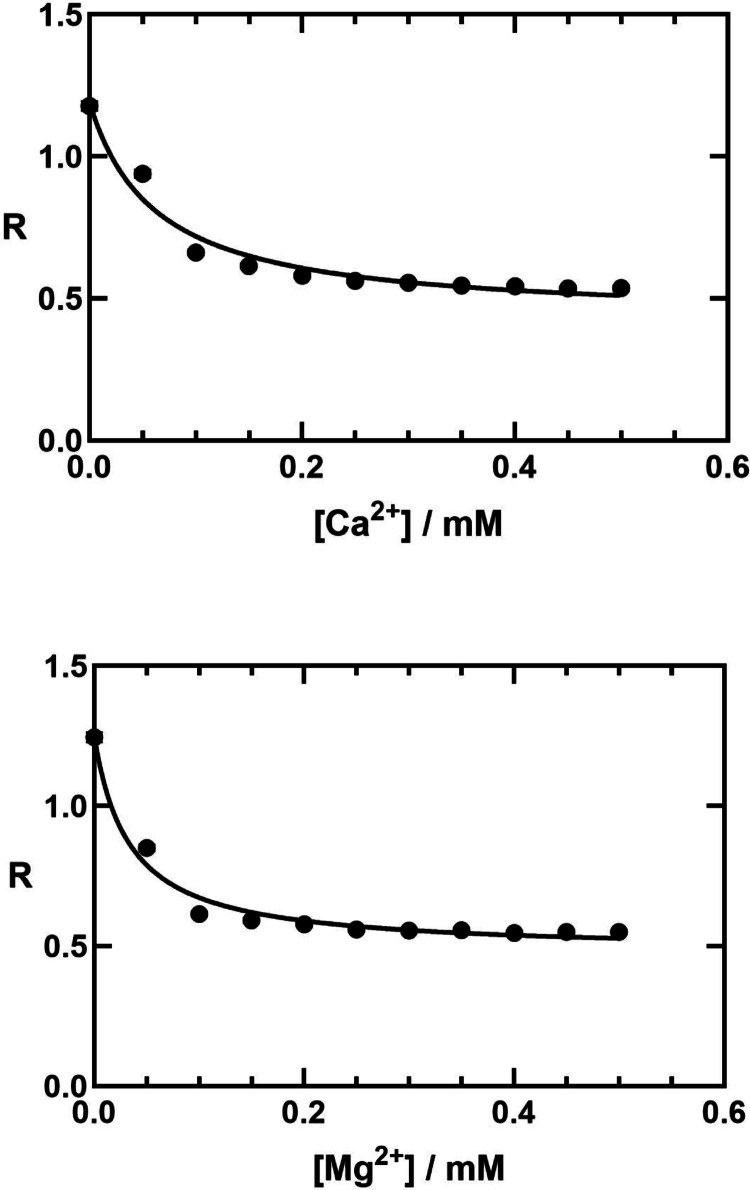
Effect of Ca^2+^ and Mg^2+^ on the fluorescence ratio, R, of eosin noncovalently bound to pig kidney Na^+^,K^+^-ATPase. R is defined as in Fig. 4. The buffer used was Tris at 1 mM and pH 7.4. The low buffer concentration was necessary to ensure that the protein was in the E2 state prior to adding either solutions of CaCl_2_ or MgCl_2_ to the cuvette. At pH 7.4, Tris, with a pK_a_ of approximately 8.1, would be expected to be 70–80% ionized. The ionic strength of the buffer would thus be 0.7–0.8 mM. Inspection of Fig. 4 shows that this value is very close to the y-intercept, where the enzyme is predominantly in the E2 state. The solid line on both curves represents a fit of the data to a hyperbolic binding equation, *R* = *R*_*0*_ + (*R*_*∞*_ - *R*_*0*_) [*c*/(*K*_*d*_ + *c*)], where *R*_*0*_ and *R*_*∞*_ are the limiting values of *R* at zero and infinite concentrations, *c*, of either CaCl_2_ or MgCl_2_, respectively, and *K*_*d*_ is the dissociation constant. For the Ca^2+^ data, the values obtained from fitting to the equation were *R*_*0*_ = 1.2 (± 0.1), *R*_*∞*_ = 0.43 (± 0.09) and *K*_*d*_ = 0.062 (± 0.037) mM. For the Mg^2+^ data, the values obtained from fitting to the equation were *R*_*0*_ = 1.3 (± 0.1), *R*_*∞*_ = 0.48 (± 0.05) and *K*_*d*_ = 0.034 (± 0.016) mM. *N* = 5

As shown in Fig. 6, both Ca^2+^ and Mg^2+^ cause pronounced shifts away from the E2 state towards the E1 state. Fitting of the observed data to hyperbolic binding curves yields half-saturating concentrations for Ca^2+^ and Mg^2+^ of 62 (± 37) µM and 34 (± 16) µM, respectively. These values equate to ionic strengths of 186 and 102 µM, which correspond to 0.19 and 0.10 mM. Comparing these values to the effect of ionic strength observed in Fig. 4, where the half-saturating ionic strength was approximately 5 mM, shows that the effects of Ca^2+^ and Mg^2+^ cannot be explained by an ionic screening effect. Ca^2+^ and Mg^2+^ must disrupt the electrostatic attraction stabilizing the E2 state by specific binding to the negatively charged partner of the electrostatic attraction.

The fact that MgCl_2_ has a significantly stronger effect than CaCl_2_ indicates that the effect is due to the cation, i.e., Mg^2+^ and Ca^2+^, not the Cl^−^ anion, because in both titrations the Cl^−^ concentration was increasing exactly in parallel. The higher affinity of the Mg^2+^ ion over that of Ca^2+^ is presumably due to the smaller size of the Mg^2+^ ion and, thus, its higher charge density. It seems reasonable to conclude, therefore, that the experimental results shown in Figs. 4 and 5 were also due to the cationic component of the ionic strength.

Control experiments were performed to check for any direct interaction between eosin and either CaCl_2_ or MgCl_2_ in aqueous solution. Up to a concentration of 0.5 mM, the highest concentration used in the titrations in the presence of the Na^+^,K^+^-ATPase, CaCl_2_ or MgCl_2_ were not found to cause any shift in the eosin fluorescence excitation spectrum. The fluorescence changes of eosin observed in the CaCl_2_ and MgCl_2_ titrations in the presence of Na^+^,K^+^-ATPase can, therefore, be confidently attributed to a change in the Na^+^,K^+^-ATPase conformational state.

### Interaction of CaCl_2_ with a Hybrid Self-assembled Membrane of the SURFE^2^R (Surface Electrogenic Event Reader)

The results presented so far indicate that the E2 state is stabilised by an electrostatic attraction which can be screened by the ionic strength of the surrounding solution and by the specific binding of either Ca^2+^ or Mg^2+^ ions to the negatively charged partner of the interaction with a micromolar affinity. However, at this stage, although we can speculate, we don’t yet know what either of the interaction partners are. All we know is that they must be relatively exposed to the surrounding solution so that they are screenable by the ionic strength of the medium.

The Surface Electrogenic Event Reader (SURFE^2^R) is an instrument designed for measuring capacitive currents due to the movement of ions onto or within membranes adsorbed to a hybrid solid supported membrane (SSM) (Garcia-Celma et al. 2007; Hussein et al. 2024; Clarke 2025). The SSM consists of a lamella of alkanethiol covalently bonded to a gold electrode surface and a lamella of lipid, generally the branched chain lipid diphytanoylphosphatidylcholine (because of its stability and lack of phase changes). Such a stable membrane model system allows fast solution exchange and supports clear measurement of transporter activation on adsorbed membrane fragments or vesicles. Here, the gold electrode surface can be considered as one plate of a capacitor, with the opposite side of the SSM (lipid layer) as a second capacitor plate. As with all capacitors, when charge builds up on one plate, this is compensated by the movement of charge on the opposite plate. Therefore, electrogenic events on the membrane, i.e., the movement of ions, are compensated by the movement of electrons to or away from the gold electrode, which allows the electrogenic events to be electrically detected. Pure capacitive currents are always transient currents, because no continuous current can pass through the dielectric medium between the plates. Therefore, the current signals measured by the SURFE^2^R are also transient, with a sharp rising phase followed by a slower decay back to zero current.

**Fig. 7:**
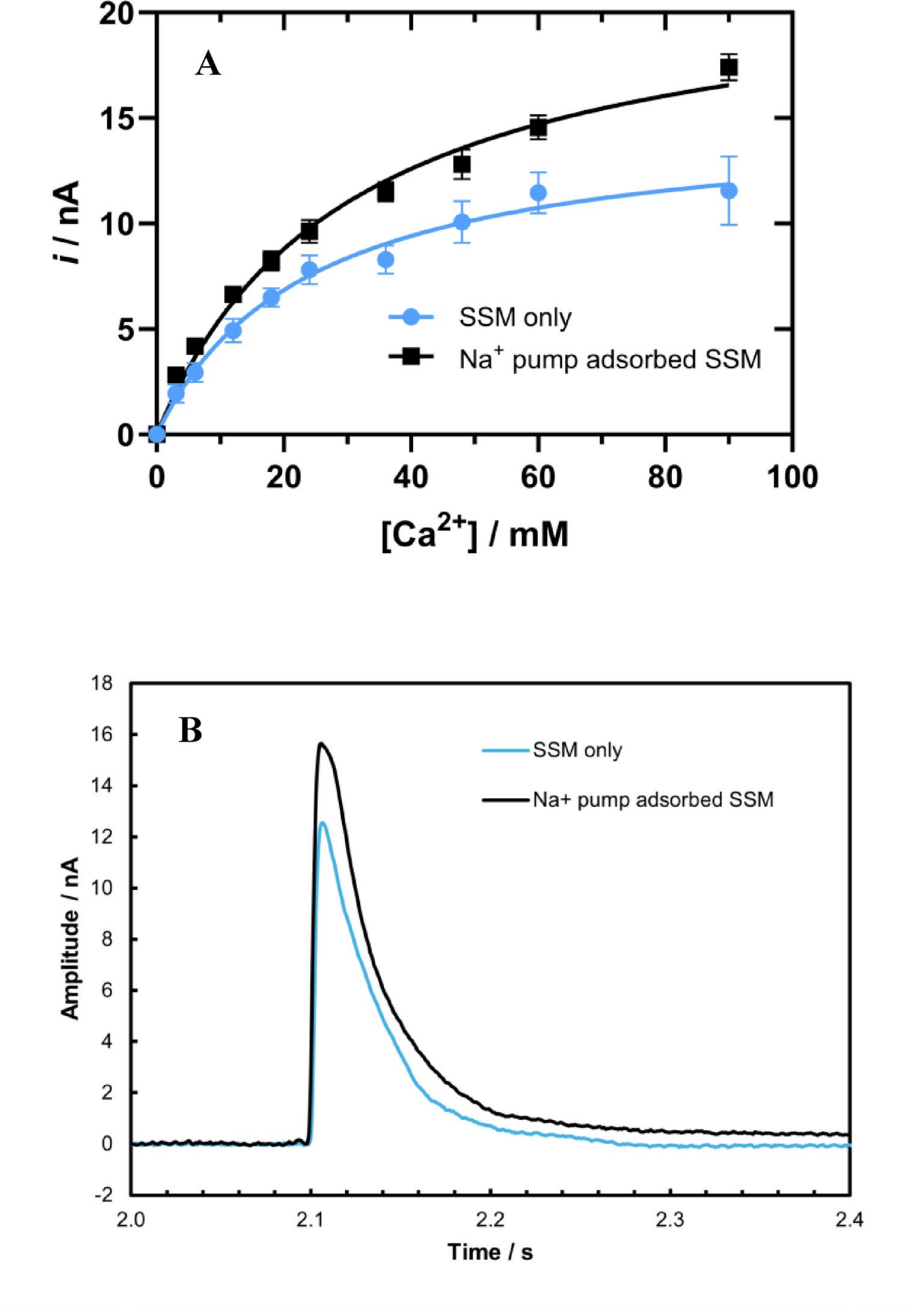
Transient currents generated by rapid flow of Ca²⁺ on an SSM alone (blue circles) and on an SSM with adsorbed Na⁺,K⁺-ATPase (black squares). Increasing Ca²⁺ concentrations led to a rise in peak current (*i*) that approached saturation (graph A), *N* = 3. Representative transient traces recorded at 60 mM Ca²⁺ highlight the difference in peak current amplitudes between the control and SSM with adsorbed Na⁺,K⁺-ATPase (graph B). The non-activating solution contained 30 mM Tris buffer and 150 mM NaCl (pH 7.4), while the activating buffer had the same composition but with CaCl_2_ added. The Ca²⁺ containing activating solution was applied to the sensor at 2.0 s, leading to interaction with the SSM and producing a transient current at 2.1 s

Using the SURFE^2^R it is possible to detect the interaction of Ca^2+^ ions with its hybrid membrane as well as with Na^+^,K^+^-ATPase-containing membrane fragments after their addition and adsorption to the SSM. Figure 7A demonstrates that in both cases, the peak current increases with rising Ca²⁺ concentrations, indicating an increase in binding, until reaching saturation at which point the SSM surface would be expected to be largely occupied by the cations. However, in the presence of membrane fragments containing adsorbed Na⁺,K⁺-ATPase, the peak currents were higher than those observed in the protein-free control. The dissociation constant K_d_ for Ca^2+^ binding to the SSM was 24 ± 11 mM, while that with Na^+^,K^+^-ATPase-containing membrane fragments adsorbed to the SSM was 30 ± 7 mM. Transient traces in Fig. 7B recorded with an activating buffer containing 60 mM Ca²⁺, further highlighted the difference in peak current amplitudes between the control and SSM with adsorbed Na⁺,K⁺-ATPase.

## Discussion

Considering the results presented here together, collected from the year 2001 to the present day, a number of conclusions seem clear. Firstly, an electrostatic attraction in the E2 state plays a key role in its stabilisation and this attraction must be broken for the enzyme to convert to the E1 state, bind Na^+^ ions, become phosphorylated by ATP and continue ion pumping. Because the E2 → E1 transition is the slowest step of the entire pump cycle for mammalian Na^+^,K^+^-ATPase, the breaking of the electrostatic attraction is crucial in determining the overall ion pumping rate and is therefore a prime site for ion pumping regulation. It is also clear that the electrostatic attraction can be broken by nonspecific screening by ionic strength or by the specific binding of calcium or magnesium ions to the negatively charged partner of the electrostatic interaction. The electrostatic attraction must be a fixed charge: fixed charge interaction, similar to a salt bridge, because hydrogen bonds are not uniformly weakened by ionic strength (Urbic 2014). Kanai et al. (2023) have shown in their crystal structure of the E2 state that in this conformation the N and A domains are tightly coupled, but in the E1 state the two domains become detached and move away from one another. Therefore, presumably the electrostatic attraction that has been identified via the results presented here must allow this detachment of the N and A domains to occur.

The SURFE^2^R results presented here show that Ca^2+^ ions can bind to lipid membranes (in agreement with previous work (Deplazes et al., 2021)) and that peak currents are higher in the presence of Na^+^,K^+^-ATPase-containing membrane fragments. A possible explanation for this is that the membrane fragments are known to contain negatively charged phosphatidylserine, to which Ca^2+^ would be expected to bind more strongly than to zwitterionic phosphatidylcholine headgroups. That the negatively charged membrane surface is an interaction partner in the electrostatic attraction is suggested by the fact that Kanai et al. (2023) could not resolve any salt bridges on the cytoplasmic side of the Na^+^,K^+^-ATPase that could stabilise the N-A domain interaction. That the other interaction partner is the N-terminus of the protein’s α-subunit is supported by the facts that this segment of the protein could not be resolved in crystal structures (Kanai et al. 2023), but it has been known for many years from tryptic digest experiments (Jørgensen and Collins 1986; Jørgensen and Andersen (1988) to undergo significant movement during the E2 → E1 transition. This is also supported by molecular dynamics simulations showing that the flexibility and length of the N-terminus allows it to interact with the membrane surface (Lev et al. 2023). It is possible that anchoring of the N-terminus onto the membrane by an electrostatic attraction in the E2 state pulls both the N and the A domains, to which the N-terminus is attached, towards the membrane surface and keeps them in a closed configuration. In the E1 state, on the other hand, when the N-terminus comes off the membrane and has more flexibility, this allows the N and A domains also more freedom of motion such that they can detach from one another.

The differences observed in the eosin measurements shown in Fig. 3 for titrations with CaCl_2_ or MgCl_2_ indicate that the shift of the Na^+^,K^+^-ATPase from E2 to E1 is due to the cation, not the anion. The *K*_*d*_ for Ca^2+^ of 62 (± 37) µM is far above the physiological level, which is approximately 0.1 µM for a resting cardiac myocyte and approximately 1 µM during a contraction (Marks 2003). Therefore, it is unlikely that the Ca^2+^ effect observed here plays a physiological role. The *K*_*d*_ for Mg^2+^ of 0.034 (± 0.016) mM is below typical physiological levels reported for free Mg^2+^ in the cytoplasm of an animal cell of 0.5–1.0 mM (Murphy 2000). However, in the cytoplasm most of the Mg^2+^ ions would be expected to be complexed by ATP. Therefore, variation in the ATP concentration could lead to changes in the free Mg^2+^ concentration and it is, thus, possible that Mg^2+^ could potentially play a regulatory role for the Na^+^,K^+^-ATPase via the interaction identified here.

## Methods

### Enzyme and Reagents

Na^+^,K^+^-ATPase-containing membrane fragments from the outer medulla of pig kidney were purified as described by Klodos et al. (2002). The activities of the preparations are typically in the range 1300–2000 µmol ATP hydrolysed h^− 1^ (mg of protein)^−1^ at saturating substrate concentrations and the protein concentration was 6 mg mL^− 1^. The protein concentration was determined according to the Peterson modification (1977) of the Lowry method (1951) using bovine serum albumin as a standard.

The origins of the various reagents used were as follows: Tris(hydroxymethyl)aminomethane (99%, Alfa Aesar, Heysham, UK), eosin Y (C.I. 45380, BDH, Kilsyth, Australia), CaCl_2_ (analytical grade, Merck, Kilsyth, Australia), MgCl_2_ (analytical grade, Merck), dioleoylphosphatidylcholine (Avanti Polar Lipids, Alabaster, AL, USA), and HCl (0.1 N Titrisol solution, Merck).

### Eosin Fluorescence Measurements

All fluorescence measurements were carried out using an RF-6000 spectrofluorophotometer (Shimadzu, Kyoto, Japan) with 1 cm pathlength quartz microcuvettes. 1000 µL of pH 7.4 Tris buffer (1.0 mM), 33 µL of Na^+^,K^+^-ATPase-containing membrane fragments (6 mg mL^− 1^) and 2.9 µL of eosin (11 µM in water) were consecutively added to the cuvette. The value of λ_em_ was 550 nm (bandwidth 5 nm) with an OG530 cut-off filter (Schott, Mainz, Germany) in front of the photomultiplier. The fluorescence ratio, *R* = *F*_490_/*F*_535_, is the ratio of the fluorescence emission recorded at each of the excitation wavelengths, 490 nm and 535 nm. At the begininning of each excitation scan, i.e., at 400 nm, a wavelength where the probe doesn’t undergo excitation, the signal was zeroed to remove any possible apparent background fluorescence. The titrations were carried out by small sequential additions of concentrated solutions of 0.1 M CaCl_2_ or MgCl_2_ in the same 1.0 mM Tris buffer.

The final Na^+^,K^+^-ATPase concentration in the cuvette was 192 µg/ml. This concentration was chosen based on previous studies by Skou and Esmann (1981, 1983) in order to saturate the eosin with protein and ensure that all of the measured fluorescence derives from protein-bound eosin and any fluorescence from eosin in the neighbouring aqueous solution can be neglected.

### SURFE^2^R Measurements

The SSM was assembled on a 3 mm gold-coated sensor chip, by first adding 50 µL of 1-octadecantethiol (0.5 mM) and left to incubate for at least 30 min in a closed Petri dish. Following this, the sensor was rinsed 3 times with isopropanol and 3 times with ultrapure water then gently dried under a stream of nitrogen. The thiolated sensor was then coated with 2.0 µL of 1,2-diphytanoyl-sn-glycero-3-phosphocholine in n-decane (7.5 mg mL^− 1^), and immediately filled with 50 µL of non-activating buffer containing 30 mM Tris and 150 mM NaCl (pH 7.4) and incubated for at least 2 h at room temperature (in a closed Petri dish with damp tissue). After the SSM was formed, 40 µL of diluted Na^+^,K^+^-ATPase-containing membrane fragments (1 mg mL^− 1^) was applied onto the SSM and centrifuged at 3,000 x g (3,000 relative centrifugal force) for 30 min at room temperature. Once the protein was immobilised on the membrane, rapid solution exchange was carried out using the SURFE²R N1 single-solution exchange workflow, with the activating buffer containing varying concentrations of Ca^2+^ (0–90 mM) in 30 mM Tris and 150 mM NaCl (pH 7.4). Peak currents and the integrated area under the curve (total charge bound) were recorded. The 0 mM Ca²⁺ traces were subtracted from all readings to remove background signal contributions. Negative control experiments were performed without the protein adsorbed to the SSM, and results compared. In every experiment, the peak currents were measured 5 times at each Ca²⁺ concentration, and each experiment was repeated across 3 independent sensors and the values averaged. Error bars on Fig. 7 indicate the standard errors.

### Tertiary Structure Analysis

The protein structure of the Na^+^,K^+^-ATPase α_1_ subunit from *Sus scrofa* was predicted by AlphaFold3 (Jumper et al. 2021), using the sequence obtained from the NCBI database (accession: NP_999414.2) (Xiong et al. 2023), with the first five propeptide residues, which are not present in the expressed protein, excluded. This model was selected to examine tertiary conformations, accounting for protein folding that may position polybasic residues in close spatial proximity or at a distance. Experimentally derived crystal structures were not utilised, because they cannot resolve the disordered N-terminal tail; this is predicted by the AlphaFold model, albeit with lower confidence in this region. However, in the 14th Critical Assessment of protein Structure Prediction (CASP14), AlphaFold models demonstrated competitive accuracy with experimentally derived structures, achieving a median backbone accuracy of 0.96 Å r.m.s.d._95_ in well-ordered regions (Jumper et al. 2021). Nevertheless, the disordered N-terminal tail should be interpreted with caution.

The porcine Na⁺,K⁺-ATPase structure (predicted by AlphaFold) was positioned and oriented in a membrane environment using the PPM 3.0 (Positioning of Proteins in Membranes) server (Lomize et al. 2022). All heteroatoms were removed to avoid bias in membrane placement. Protein topology was adjusted to ensure consistency with the experimentally established organization of cytoplasmic and extracellular domains of Na⁺,K⁺-ATPase. Calculations were performed assuming a flat mammalian plasma membrane (without curvature) with a predefined hydrophobic thickness (D_0_) of 32.0 Å and a membrane stretching stiffness (f_mism_) of 0.02 kcal mol⁻¹·Å⁻². The optimal embedding was characterized by an effective hydrophobic thickness of 31.4 ± 0.5 Å and a highly favourable transfer free energy (ΔG_transfer_) of − 49.2 kcal·mol⁻¹. The resulting tilt angle was approximately 29° relative to the membrane normal, indicating a vertical insertion profile. The final membrane-coordinated structure was exported in PDB format for subsequent visualization and computational analysis.

Electrostatic potential maps were generated using the Adaptive Poisson-Boltzmann Solver (APBS) (version 3.4.1) with the automatic multigrid focusing method (mg-auto) (Jurrus et al. 2018) based on the Na⁺,K⁺-ATPase structure obtained from PPM 3.0. Prior to APBS calculations, atomic charges, radii, and protonation states were assigned using the PDB to PQR file conversion server, PDB2PQR (version 3.7.1) (Dolinsky et al. 2007). The PPM-oriented PDB structure was processed under aqueous conditions at pH 7.0, applying default PDB2PQR algorithms, including steric clash removal, hydrogen-bond network optimization, and pH-dependent titration state assignment using the Protein pKa (PROPKA) server (Li et al. 2005). Protonation states were assigned to titratable residues based on PROPKA predictions, restricted by the Parameters for Solvation Energy (PARSE) force field (Sitkoff et al. 1994) compatibility; standard ionization states were retained for residues where specific protonation variants were not parameterized. Electrostatic potentials were computed by solving the linearized Poisson–Boltzmann equation using a solute (i.e., protein) dielectric constant of 2 and a solvent dielectric constant of 78.54 corresponding to water, with the temperature set to 298.15 K. The solvent was modeled with an ionic strength of 0.15 M, represented by two monovalent ion species (± 1e charge) each at 0.15 M concentration and an effective ionic radius of 2.0 Å. The resulting electrostatic potentials were generated as DX files and subsequently visualized by mapping the calculated potentials onto membrane-oriented AlphaFold-derived protein structures using PyMOL (PyMOL Molecular Graphics System, version 3.1.6.1; Schrödinger, LLC). Electrostatic surfaces were rendered using a red-white-blue colour ramp spanning − 10 to + 10 kT/e, applied via PyMOL surface colouring commands to enable direct visualization of charge distributions across the protein surface.

## Data Availability

The authors declare that the main data supporting the findings of this study are available within the article.
